# Ultralight metal foams

**DOI:** 10.1038/srep13825

**Published:** 2015-09-08

**Authors:** Bin Jiang, Chunnian He, Naiqin Zhao, Philip Nash, Chunsheng Shi, Zejun Wang

**Affiliations:** 1 School of Materials Science and Engineering, Tianjin University, Tianjin 300072, P.R. China; 2 Tianjin Special Equipment Inspection Institute, Tianjin 300192, P.R. China; 3 Thermal Processing Technology Center, Illinois Institute of Technology, Chicago, IL 60616, USA

## Abstract

Ultralight (<10 mg/cm^3^) cellular materials are desirable for thermal insulation; battery electrodes; catalyst supports; and acoustic, vibration, or shock energy damping. However, most of these ultralight materials, especially ultralight metal foams, are fabricated using either expensive materials or complicated procedures, which greatly limit their large-scale production and practical applications. Here we report a simple and versatile method to obtain ultralight monolithic metal foams. These materials are fabricated with a low-cost polymeric template and the method is based on the traditional silver mirror reaction and electroless plating. We have produced ultralight monolithic metal foams, such as silver, nickel, cobalt, and copper via this method. The resultant ultralight monolithic metal foams have remarkably low densities down to 7.4 mg/cm^3^ or 99.9% porosity. The metal foams have a long flat stress-train curve in compression tests and the densification strain ε_D_ of the Ni/Ag foam with a porosity of 99.8% can reach 82%. The plateau stress σ_pl_ was measured and found to be in agreement with the value predicted by the cellular solids theory.

Ultralow-density (<10 mg/cm^3^) foams are a relatively new class of materials with a unique combination of properties such as low density, gas permeability, and thermal conductivity. Therefore, these materials show potential to enable new technologies in areas as diverse as catalysis, fuel cells, hydrogen storage, and acoustical insulation. Currently, very few materials exist in the ultralight regime below 10 mg/cm^3^: carbon aerogels (density ρ ≥ 0.16 mg/cm^3^)^1^, silica aerogels (ρ ≥ 1 mg/cm^3^)^2^, carbon nanotube aerogels (ρ ≥ 5 mg/cm^3^)^3^,^4^, graphene aerogels (ρ ≥ 3 mg/cm^3^)^5^, metallic foams (ρ = 0.9–55 mg/cm^3^)^6^,^7^,^8^, and polymer foams (ρ = 8–10 mg/cm^3^)^9^. However, most of these ultralight materials were fabricated using either expensive materials or complicated procedures, which greatly limit their large-scale production and practical applications. In addition, the ability to form ultralight monolithic metal foams is more difficult because of the high densities of metals, and approaches to the synthesis of ultralight non-metallic foams are not well-suited for metals. Thus far, there are few well-established approaches for preparing ultralight monolithic metal foams. Ultralight magnetic Ni/C, Co/C, and Fe_2_O_3_/C foams were fabricated on the centimeter scale by pyrolyzing commercial polyurethane sponge grafted with polyelectrolyte layers based on the corresponding metal acrylate^8^. B.C. Tappan *et al.* reported a relatively simple method for obtaining ultralow-density, monolithic, transition-metal foams (iron, cobalt, copper), utilizing self-propagating combustion synthesis of novel transition-metal complexes containing high nitrogen energetic ligands^7^. However, the metal foams were not pure metal or alloy, with the remainder composed of varying amounts of carbon. Ultralow-density (<10 mg/cm^3^) pure metal or alloy foams have only been reported by T.A. Schaedler^6^. An ultralight nickel phosphorus alloy based on periodic hollow-tube microlattices was fabricated by starting with a template formed by self-propagating photopolymer waveguide prototyping, coating the template by electroless nickel plating, and subsequently etching away the template. The resulting metallic microlattices exhibit ultralow density (0.9 mg/cm^3^). Distinct from other metal foams, the metallic hollow-tube lattice shows complete recovery after compression exceeding 50% strain and energy absorption similar to elastomers.

Until now, developing a simple and versatile method for the synthesis of ultralight monolithic metal foams still remains a great challenge. Herein, we report a novel and facile method to fabricate ultralight monolithic metal foams, such as silver, nickel, cobalt, and copper. These as-made monolithic metal foams have remarkably low densities down to 7.4 mg/cm^3^ or 99.9% porosity.

## Results and Discussion

**Figure 1:**
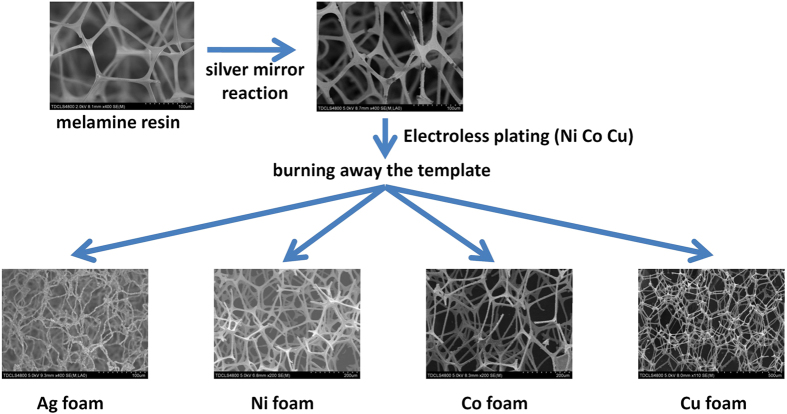
Fabrication scheme of ultralight metal foams.

Figure 1 illustrates the fabrication scheme of the ultralight Ag, Ni, Co, Cu foams. These materials are prepared by starting with a polymeric template, coating the template via the silver mirror reaction, then electroless plating (Ni, Co, Cu), and subsequently burning away the template. The template employed in this study is a polymer foam used as a household cleaning eraser which is cheap and easy to purchase in the market. The polymer made from melamine resin is flexible with a three-dimensional network structure consisting of slender filaments (Supplementary Fig. S1). The melamine resin foam is an extremely open-cell foam with highly hydrophilic properties, which are advantageous for the electroless plating. The traditional method of electroless plating on dielectric templates such as a polymer involves two, often three, stages^10^. The polymer templates must be catalytically activated prior to the electoless plating to provide a surface that can interact with metal ions in solution causing their reduction on the surface and growth of the coating. SnCl_2_ and PdCl_2_ are most commonly used as activators for this purpose^11^,^12^,^13^. However, PdCl_2_ is expensive and toxic. In our study, a traditional and practical method, the silver mirror reaction, was successfully used to fabricate continuous silver films in three-dimension on the polymer template as the catalytic activator prior to the electroless plating (Ni, Co, Cu).

**Figure 2:**
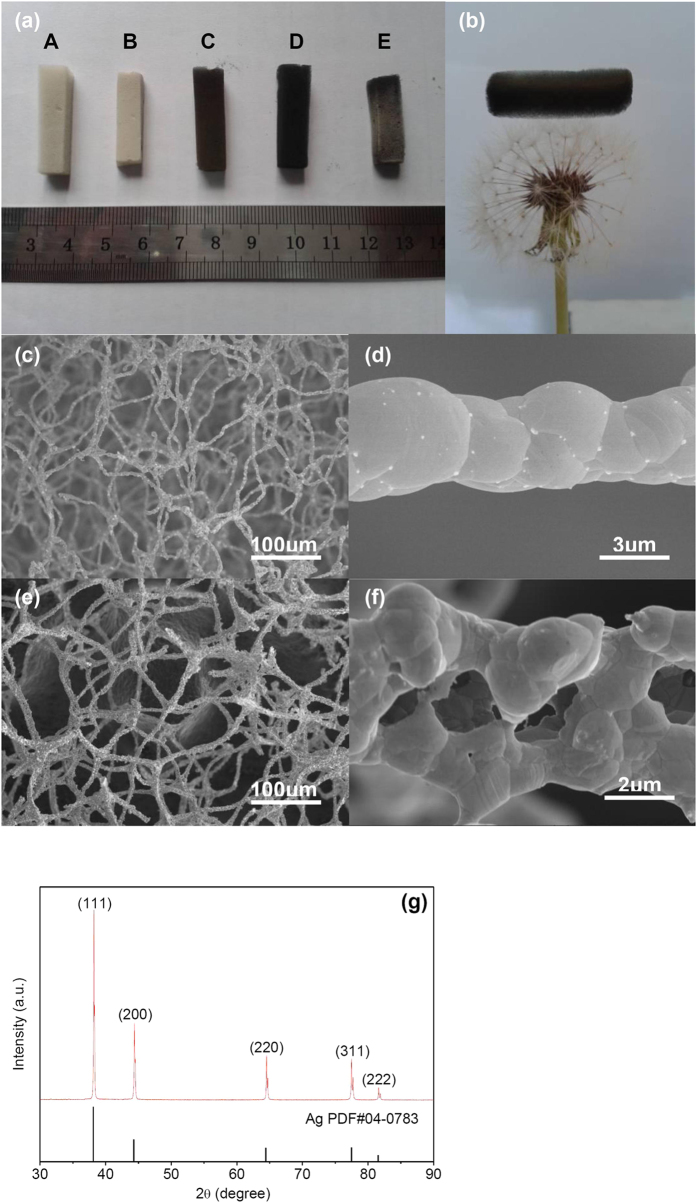
Macroscopic and microscopic structures of ultralight metal foams. (**a**) Digital photograph of ultralight metal foams: (A) the polymer template acquired after the silver mirror reaction; (B) Ag foam; (C) Ni foam; (D) Co foam; (E) Cu foam. (**b**) A piece of ultralight Ni foam with the density of 7.4 mg/cm^3^ supported on a dandelion. (**c**) Low-magnification SEM image of the Ag foam. (**d**) Highly magnified image of a filament of the Ag foam. (**e**) SEM image of the Ag foam with hollow filaments. (**f**) The hollow filament of Ag foam. (**g**) XRD patterns of the Ag foam.

Figure 2a shows the digital photograph of ultralight metal foams (Ag, Ni, Co, Cu) and the polymer template acquired after the silver mirror reaction. A piece of ultralight Ni foam with the density of 7.4 mg/cm^3^ is shown supported on a dandelion (Fig. 2b). The polymer foam with Ag coating acquired after the silver mirror reaction was heated to 700 °C in an air atmosphere in a muffle furnace to burn away the polymer template, and then, ultralight monolithic Ag foams were obtained (Fig. 2a (B)). The porosity of this ultralight monolithic Ag foam is 99.8% (ρ = 18.7 mg/cm^3^, 11.8 mg in 0.63 cm^3^). The heating process results in an obvious decrease in volume (by roughly 50%) from the polymer foam image, as shown in Fig. 2a (A) and (B). A low-magnification SEM image of the monolithic Ag foam (Fig. 2c) shows a three-dimensional network structure consisting of uniform slender filaments. The filaments of the Ag foam became curled (Supplementary Fig. S2) during the heating process which results in a dramatic decrease in volume of the Ag foam. Figure 2d shows a highly magnified image of the morphology of a filament of the Ag foam, which is analogous to the silver sponges reported in the literature^14^. The filament is approximately 3 μm in diameter and is produced from the coarsening of interconnected silver particles. Energy dispersive spectrometer (EDS) analysis of the silver filament showed that only silver is present (Supplementary Fig. S3). In X-ray diffraction (XRD), strong metallic silver reflections were observed from an Ag foam specimen, and no other residual peaks were detected (Fig. 2g). It is noticed that the microstructure of the Ag foam can be tuned by altering the heating temperature. By reducing the heating temperature from 700 °C to 680 °C, hollow filaments were obtained in the Ag foam (Fig. 2e,f). The filament is tubular in structure, and many holes exist in the wall.

**Figure 3:**
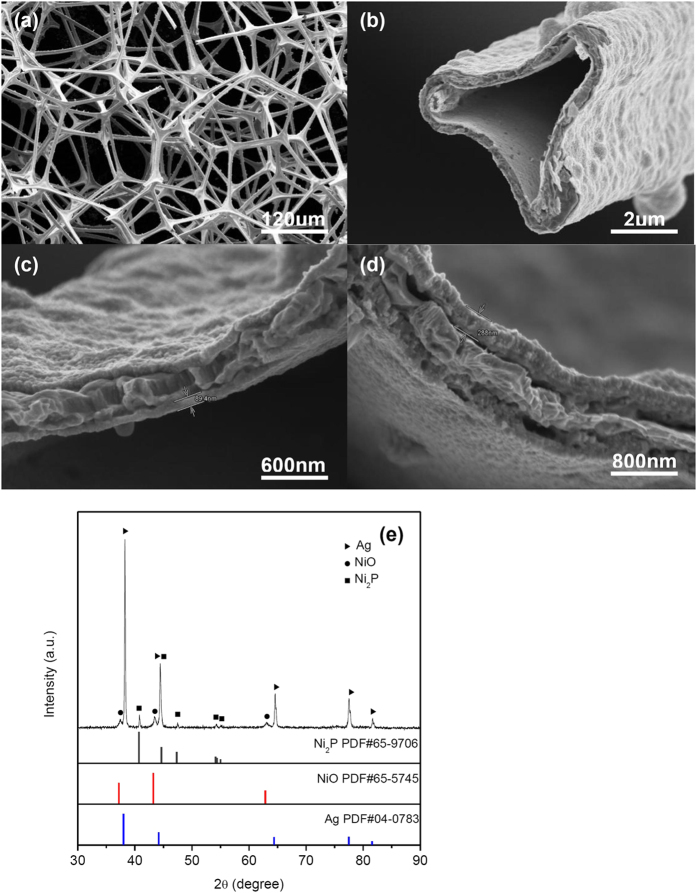
Microscopic structures of ultralight Ni/Ag foams. (**a**) Low-magnification image of the Ni/Ag foam. (**b**) SEM image of a filament of the Ni/Ag foam. (**c**) and (**d**) the microscopic structures of the film of the ultralight Ni/Ag foam. (**e**) XRD patterns of the Ni/Ag foam.

As presented above, different Ag foam microstructures were obtained as the heating temperature was changed. Since the polymer used in our study only completely decomposed at a temperature >680 °C, it was not possible to use a lower temperature. The microstructure of the Ag foam should be similar with the original polymer foam template (Supplementary Fig. S1). The initial silver film is about 200–300 nm in thickness (Fig. 3c). The wall thickness of the Ag tube should be 200–300 nm. Corresponding SEM studies (Fig. 2e,f) indicated that the change in morphology was associated with increased consolidation of the framework due to coarsening of the interconnected silver particles, growth of sintering necks, and reduction in size of the void spaces. When the foam was heat-treated at 680 °C in the muffle furnace to burn away the polymer template, the particle size of the silver film which is 100–200 nm obtained by the silver mirror reaction increased to 500–2000 nm (Supplementary Fig. S4). Many holes on the silver film were produced due to decomposition and gasification of the polymer substrate at high temperatures. The coarsened silver film composed of a tubular structure can stand as a monolithic structure on its own, in isolation from the template. As the heating temperature was increased to 700 °C, the particles of the silver film continuously coarsened. The tubular structure of the silver film transformed to a solid structure in order to reduce the surface energy, with the particles growing to 2–3 μm. The filaments of the Ag foam became curled during the heating process (Supplementary Fig. S2). This was most prominent for samples prepared at 750 °C. The filaments of the Ag foam became so soft and flexible that they could not bear their own weight. The shape of the foam became irregular and the dimensions decreased greatly.

After the silver mirror reaction, the polymer template was coated by electroless nickel plating and subsequently the template was burned away in a muffle furnace, then, ultralight monolithic Ni/Ag foams were acquired (Fig. 3a). The porosity of this ultralight monolithic Ni/Ag foam can reach 99.9% (ρ = 7.4 mg/cm^3^, 13.4 mg in 1.79 cm^3^). The foam has a three-dimensional network structure consisting of uniform slender filaments, analogous to the original polymer foam template (Supplementary Fig. S1). Figure 3b shows that the filament is tubular in structure and the cross section of the filament of the Ni/Ag foams is triangular which is similar to the filament of the original polymer foam template. Compared with the Ag foam, the metal films of the Ni/Ag foam did not change in morphology during burning away the polymer template. The Ni/Ag films had enough strength and were not damaged when the polymer substrate decomposed and gasified at high temperatures. The structure of the film of the Ni/Ag foam is shown in Fig. 3c. The film is a sandwich structure (Ni/Ag/Ni) and the Ni layer is only 89 nm in thickness. The thickness of the Ni layer can easily be controlled by changing the time of the electroless nickel plating (Fig. 3d) and the Ni/Ag foams with different densities can be produced. The Ni/Ag foam should form a double layer structure in theory. But all the Ni/Ag foams synthesized by the method form a sandwich structure (Ni/Ag/Ni). The adhesion between the silver film and the polymer is not good and there is small interstice between the silver film and the polymer (Supplementary Fig. S5). The driving force (the potential difference) of electroless nickel plating is higher than that of other metals. During electroless nickel plating, the nickel film was produced rapidly with a lot of gas (H_2_) releasing on the surface of the silver film. The electroless nickel plating solution can enter the interstice between the silver film and the polymer continuously under the agitation of the gas. Then the nickel film on the inner surface of silver film was synthesized. So the Ni/Ag foams synthesized by the method form a sandwich structure (Ni/Ag/Ni). The EDS analysis of the foam showed that silver, nickel, phosphorus and oxygen are present (Supplementary Fig. S6). In XRD, Ag, NiO and Ni_2_P peaks were observed from a Ni/Ag foam specimen (Fig. 3e). In the sandwich structure, the Ag coating was produced by the silver mirror reaction and the Ni films should remain as a supersaturated solid solution of phosphorous in a crystalline face-centered cubic nickel lattice after deposition^6^. Because the foam was heated at 700 °C in air in order to remove the polymer template and the Ni film is only about 100 nm thick, the nickel was all oxidized to generate NiO with Ni_2_P precipitates present and no peaks of any residual nickel were detected in the XRD pattern.

Similarly, the polymer foam acquired after the silver mirror reaction was coated by electroless cobalt or copper plating and subsequently burning away the polymer template, then, ultralight monolithic Co/Ag, Cu/Ag foams were synthesized (Supplementary Fig. S7). EDS analysis of the Co/Ag foam showed that silver, cobalt and phosphorus are present. The film is a double layer structure (Co/Ag) and the thickness of the Co layer can also be easily controlled by changing the time of electroless plating. The copper particles of the Cu/Ag foam are coarse and no phosphorus exists in the metal film. A Ni foam without Ag can be produced by immersing the Ni/Ag foam into a solution of hydrogen peroxide and ammonia. The SEM image and EDS of the Ni foam are shown in Supplementary Fig. S7. When the silver film was dissolved in the hydrogen peroxide solution, the reaction was violent and a lot of gases emerged. Therefore, the nickel films with a little remainder amounts of Ag were oxidized and damaged, resulting in the irregular shape of the ultralight monolithic Ni foams. The strength of the monolithic Ni foams was not enough to perform the compression tests. So removal of Ag from the Co/Ag foam and Cu/Ag foam were not implemented.

**Figure 4:**
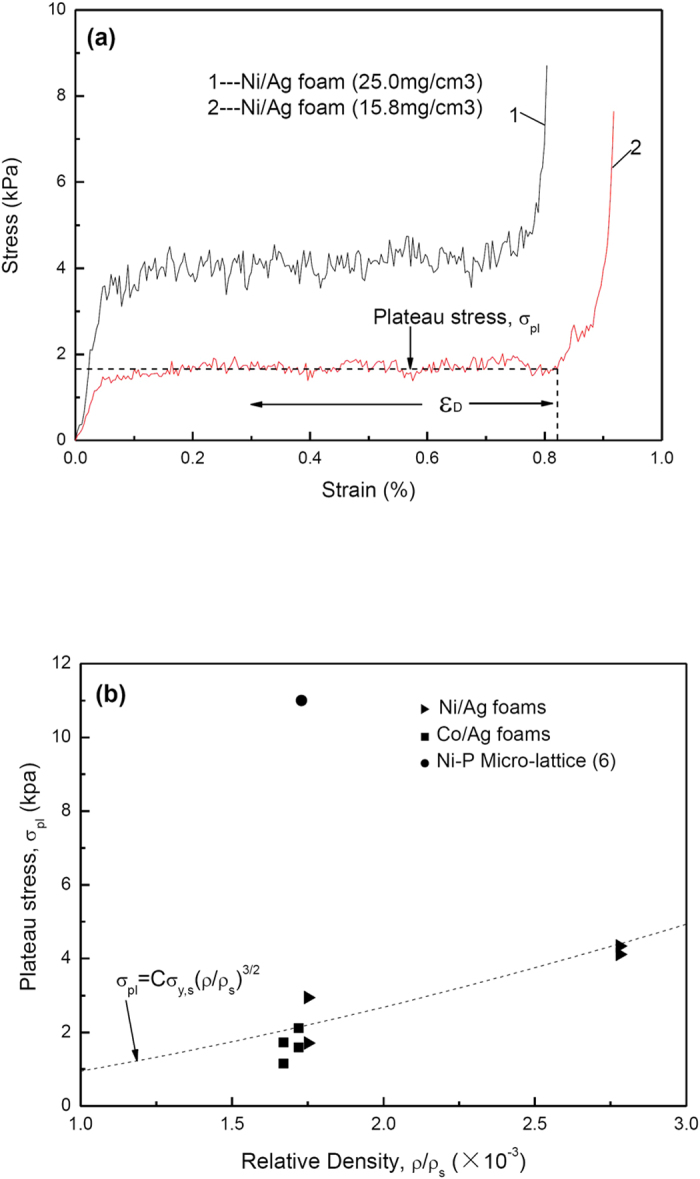
Compression test results of the ultralight metal foams. (**a**) Stress-strain curves of Ni/Ag foams with different densities. (**b**) Plateau stress of metal foams at low relative density.

The compression test results of the ultralight metal foams are shown in Fig. 4. The present Ni/Ag foam shows a similar stress–strain behavior compared with the other metallic foams^15^,^16^, characterized by three distinct regions, i.e. linear elastic deformation, collapse plateau and densification region (Fig. 4a). Ideal energy absorbers have a long flat stress-train curve. The absorber collapses plastically at a constant nominal stress, called the plateau stress, σ_pl_, up to a limiting nominal strain, ε_D_. Energy absorbers for packaging and protection are chosen so that the plateau stress is just below that which will cause damage to the packaged object. The best choice is then the one which has the longest plateau, and therefore absorbs the most energy before reaching ε_D_. The area under the curve, roughly σ_pl_ε_D_, measures the energy the foam can absorb, up to the end of the plateau. Foams which have a long flat stress-strain curve perform well in this function. Hollow tubes, shells, and metal honeycombs have the appropriate type of stress-strain curves^17^. All the Ni/Ag and Ni/Co foams have hollow tubes, so the densification strain ε_D_ of the Ni/Ag foam with the porosity of 99.8% (density 15.8 mg/cm^3^) can reach 82%. The relatively brittle nature of the electroless nickel thin film result in the collapse plateau being a serrated curve and the Ni/Ag foam sample became powders after the compression test. The energy per unit volume absorbed by the foam up to densification is 1.45 mJ/cm^3^. A stress-strain curve of a Ni/Ag foam with a higher density (25.0 mg/cm^3^) is also shown in Fig. 4a. With increasing density of the foam, the plateau stress σ_pl_ increased and the densification strain ε_D_ decreased. The energy per unit volume absorbed by this foam is 3.29 mJ/cm^3^.

The relationships among the plateau stress of the foam, the yield strength of the cell wall material and the relative density, ρ*/ρ, have been reported by M.F. Ashby^17^. For open-celled material, the relationship can be determined in terms of the following equation


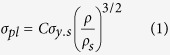


where σ_pl_ is the collapse plateau stress, σ_y.s_ is the yield stress of the cell wall material, ρ is the density of the foam and ρ_s_ is the density of the cell wall material, respectively. C is a constant between 0.25 and 0.35. M.F. Ashby showed that the value of C is 0.3 from data of cellular metals. Nominal yield strength for the fully dense pure silver is: σ_y.s _= 35 Mpa^18^. The σ_y.s_ of the electroless nickel thin film is 150–200 Mpa^18^. The σ_y.s_ of the cell wall material (Ni/Ag/Ni thin films) is chosen to be 100 Mpa. According to equation (1), the theoretical plateau stress σ_pl_ with different relative densities are shown in Fig. 4b (the dotted line). The experimental plateau stress of the Ni/Ag foams and Co/Ag foams are also marked. The average of the stress when the strain is from 0.1 to ε_D_ is taken as the value of the plateau stress σ_pl_. There is agreement with the experimental datas with the theoretical plateau stress. With the similar relative density, the Ni-P microlattices^6^ have a higher plateau stress (Fig. 4b) than the foams of our study. The base architecture of Ni-P microlattices consists of a periodic array of hollow tubes that connect at nodes, forming a hierarchical cellular architecture at three distinct length scales. So the Ni-P microlattices exhibit a higher plateau stress and energy absorption, even the entirely different mechanical properties . On the other hand, the cell wall of the foams in our study contains a lot of pure silver resulting in the yield stress of the cell wall being dramatically lower.

In summary, our results illustrate a simple and versatile method to obtain ultralight monolithic metal foams. These materials are fabricated with a low-cost polymeric template and the method based on the traditional silver mirror reaction and electroless plating. We have produced ultralight monolithic metal foams, such as silver, nickel, cobalt, and copper via this method. The resultant ultralight monolithic metal foams have remarkably low densities down to 7.4 mg/cm^3^ or 99.9% porosity. The densities of the metal foams can be easily controlled by changing the time of electroless plating. The metal foams have a long flat stress-train curve in the compression test and the densification strain ε_D_ of the Ni/Ag foam with a porosity of 99.8% can reach 82%. The plateau stress σ_pl_ was measured and found to be in agreement with the value predicted by the cellular solids theory. We expect to be able to apply this method to many other metals and anticipate some possible applications for an ultralight material with energy absorption.

## Methods

### Materials

The polymer foam was obtained from B. A. S. F. Co. Ltd. All the chemicals were bought from Tianjin Chemicals Co. Ltd. and used as received.

### The silver mirror reaction method

Polymer foam with dimensions of 8 mm × 8 mm × 30 mm was cut, and no extra pretreatments for the foam were performed prior to use. The silver mirror reaction bath contained silver nitrate, ammonia and glucose. First, an ammonia solution (2 wt%) was added dropwise to 10 ml silver nitrate solution (2 wt%) until the precipitate completely dissolved to form [Ag(NH_3_)_2_]+. Then, 5 ml of glucose solution (10 wt%) was added. The polymer foam sample was immediately dipped into the silver mirror reaction bath for 10 minutes at 50 °C. Subsequently, the sample was rinsed in distilled water and completely dried in a drier at 120 °C for 30 minutes.

### Synthesis of ultralight silver foams

The polymer foam acquired after the silver mirror reaction was heated to 700 °C in an air atmosphere in a muffle furnace to burn away the polymer template, and the ultralight monolithic silver foams were synthesized.

### Synthesis of ultralight Ni/Ag foams

The polymer foam acquired after the silver mirror reaction was coated by electroless nickel plating. The samples were immersed in electroless nickel plating solution with nickel sulfate (40 g/L) as the nickel source, sodium hypophosphite (40 g/L) as a reducing agent, and sodium citrate (30 g/L) and acetic acid as complexing agents. The electroless nickel plating bath was kept at pH 4.9 and plating was performed at 90 °C. A wall thickness *t* of 100 nm was achieved by electroless nickel plating of approximately 5 minutes. Subsequently, the sample was rinsed in distilled water and completely dried in a drier at 120 °C for 30 minutes. Finally, the sample was heated to 700 °C in an air atmosphere in a muffle furnace to burn away the polymer template, and the ultralight monolithic Ni/Ag foams were synthesized. Samples with a wall thickness (Ni) below ~100 nm could not remain the original size and shrank similar to the silver foam after burning away the polymer template.

### Synthesis of ultralight Co/Ag foams

The polymer foam acquired after the silver mirror reaction was coated by electroless cobalt plating. The samples were immersed in electroless cobalt plating solution with cobalt sulfate (14 g/L) as the cobalt source, sodium hypophosphite (21 g/L) as the reducing agent, and sodium citrate (60 g/L) and boracic acid (30 g/L) as complexing agents. The electroless cobalt plating bath was kept at pH 8.0 by addition of sodium hydroxide and plating was performed at 90 °C. Subsequently, the sample was rinsed in distilled water and completely dried in a drier at 120 °C for 30 minutes. Finally, the sample was heated to 700 °C in an air atmosphere in a muffle furnace to burn away the polymer template, and the ultralight monolithic Co/Ag foams were synthesized.

### Synthesis of ultralight Cu/Ag foams

The polymer foam acquired after the silver mirror reaction was coated by electroless copper plating. The samples were immersed in electroless copper plating solution with copper sulfate (6 g/L) as the copper source, sodium hypophosphite (28 g/L) as the reducing agent, and sodium citrate (15 g/L) and boracic acid (30 g/L) as complexing agents. The electroless copper plating bath was kept at pH 8.2 by addition of sodium hydroxide and plating was performed at 65 °C. Subsequently, the sample was rinsed in distilled water and completely dried in a drier at 120 °C for 30 minutes. Finally, the sample was heated to 700 °C in an air atmosphere in a muffle furnace to burn away the polymer template, and the ultralight monolithic Cu/Ag foams were synthesized.

### Synthesis of ultralight Ni foams

The Ni/Ag foam samples were immersed in an aqueous solution of hydrogen peroxide (15 wt%) and ammonia (12.5 wt%) for 30 minutes. Subsequently, the sample was rinsed in distilled water and completely dried in a drier at 120 °C for 30 minutes. The ultralight monolithic Ni foams were synthesized.

### Characterization

The foam dimensions and weight were measured with a caliper (Tianjin Measure Corp.) with an accuracy of 0.02 mm and a balance (Sartorius BSA124S) with an accuracy of 0.1 mg. The density is calculated by using the weight of the solid structure but not including the weight of air in the pores. Scanning electron microscopy (SEM) and energy dispersive spectrometry (EDS) of the polymer foam and the metal foams were performed by using a field-emission scanning electron microscope (Hitachi S-4800). X-ray diffraction (XRD) patterns were taken by a Bruker D/max 2500 v/pc X-ray diffractometer using Cu Ka radiation (k = 0.15 nm) at a scanning rate of 8.0^0^/min, using a voltage of 40 kV and a current of 200 mA. Ni/Ag foams and Co/Ag foams with dimensions of 8 mm × 8 mm × 14 mm were prepared for the compression tests. The compression tests were performed on a servo-electric Instron 5848. The displacement rate was accurately controlled at 0.05 mm/s. The loads were measured using a load cell with a capacity of 5N, and the displacements were measured by the internal displacement sensor.

## Additional Information

**How to cite this article**: Jiang, B. *et al.* Ultralight metal foams. *Sci. Rep.* **5**, 13825; doi: 10.1038/srep13825 (2015).

## Supplementary Material

Supplementary Information
