# Supplementary material for: Ultralight metal foams
Source: Sci Rep. 2015 Sep 8;5:13825. doi: 10.1038/srep13825 (PMC4562267; doi:10.1038/srep13825)
Supplement: Supplementary Information [file srep13825-s1.pdf]

Supplementary Information

Ultralight metal foams

Bin Jiang[1,2], Chunnian He[1], Naiqin Zhao[1,*], Philip Nash[3], Chunsheng Shi[1], Zejun Wang[2]

[1]School of Materials Science and Engineering, Tianjin University, Tianjin 300072, P.R. China.

[2]Tianjin Special Equipment Inspection Institute, Tianjin 300192, P.R. China.

[3]Thermal Processing Technology Center, Illinois Institute of Technology, Chicago, IL 60616, USA.

Correspondence and requests for materials should be addressed to N. Z. (nqzhao@tju.edu.cn)

Supplementary Figure S1

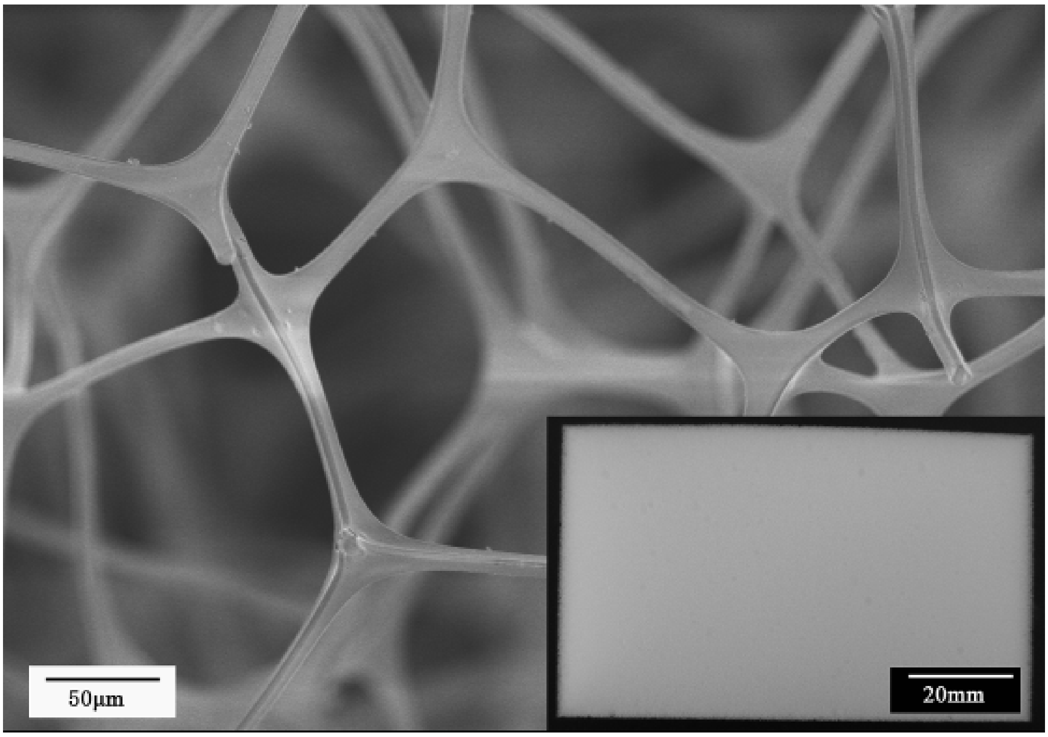

Figure S1. SEM image and optical micrograph (inset) of the polymer foam.

Supplementary Figure S2

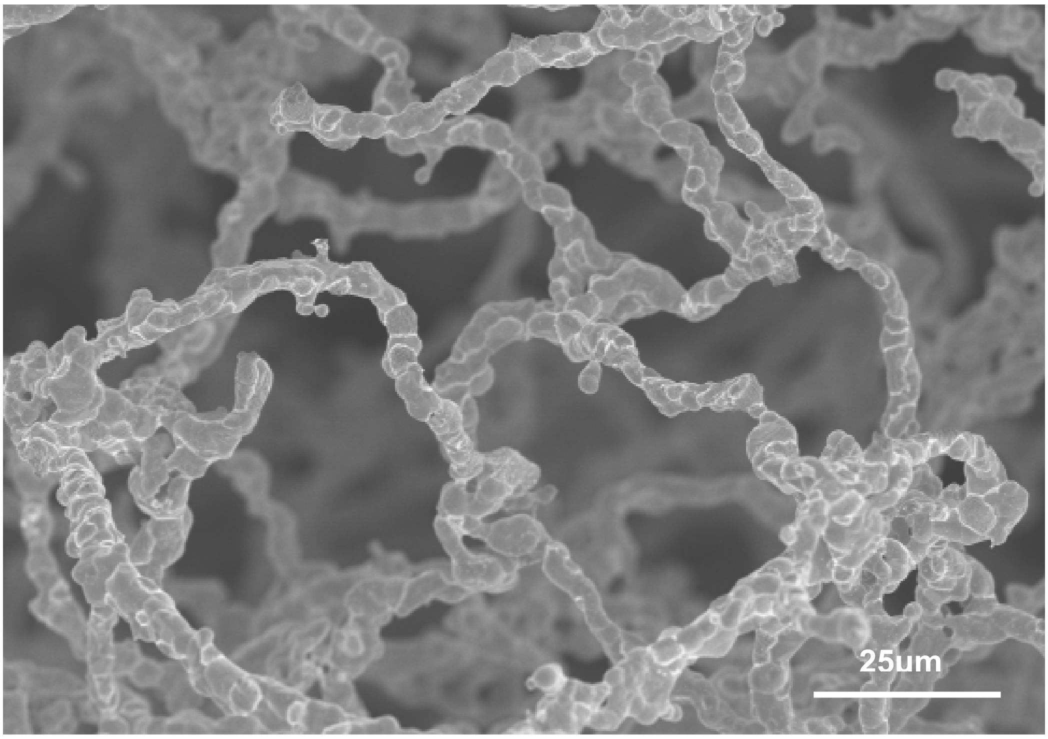

Figure S2. The curled filaments of the Ag foam.

Supplementary Figure S3

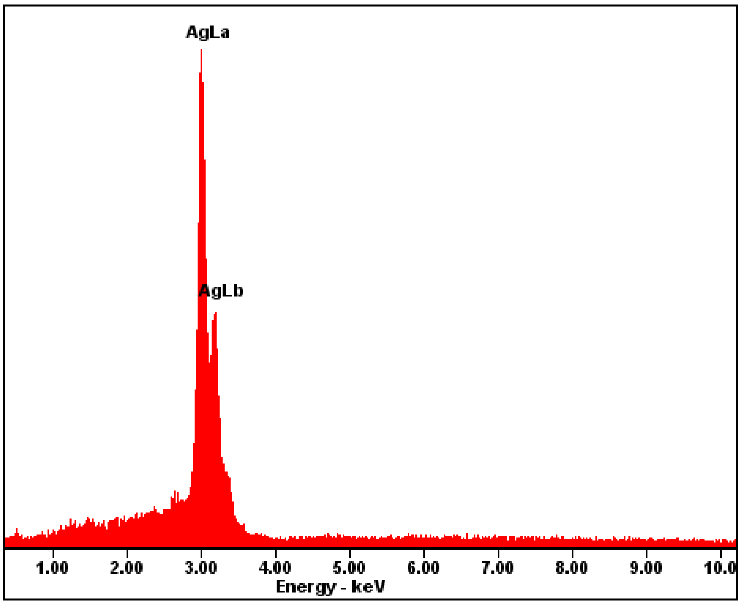

Figure S3. Energy dispersive spectrometer (EDS) analysis of the silver filament.

Supplementary Figure S4

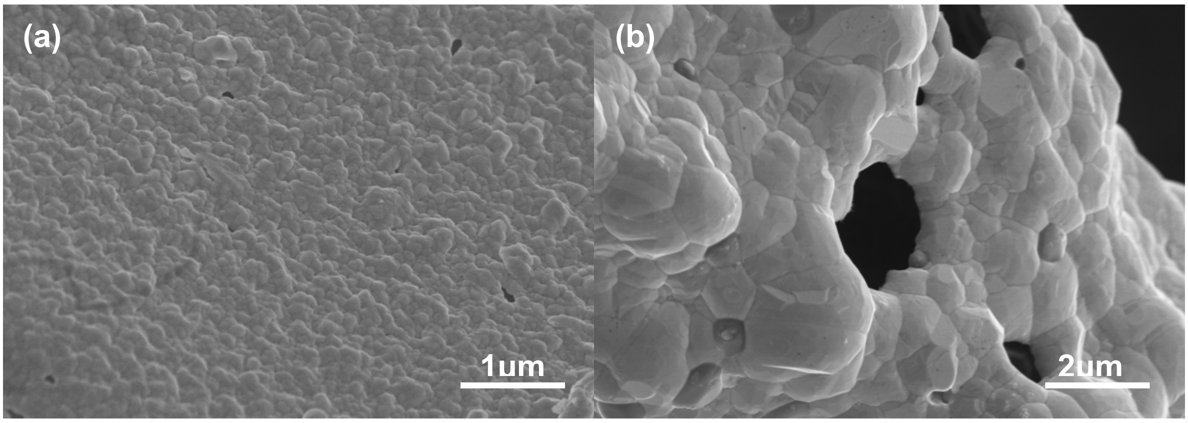

Figure S4. (a) The silver film obtained by the silver mirror reaction. (**b**) The wall of the hollow filament of Ag foam.

Supplementary Figure S5

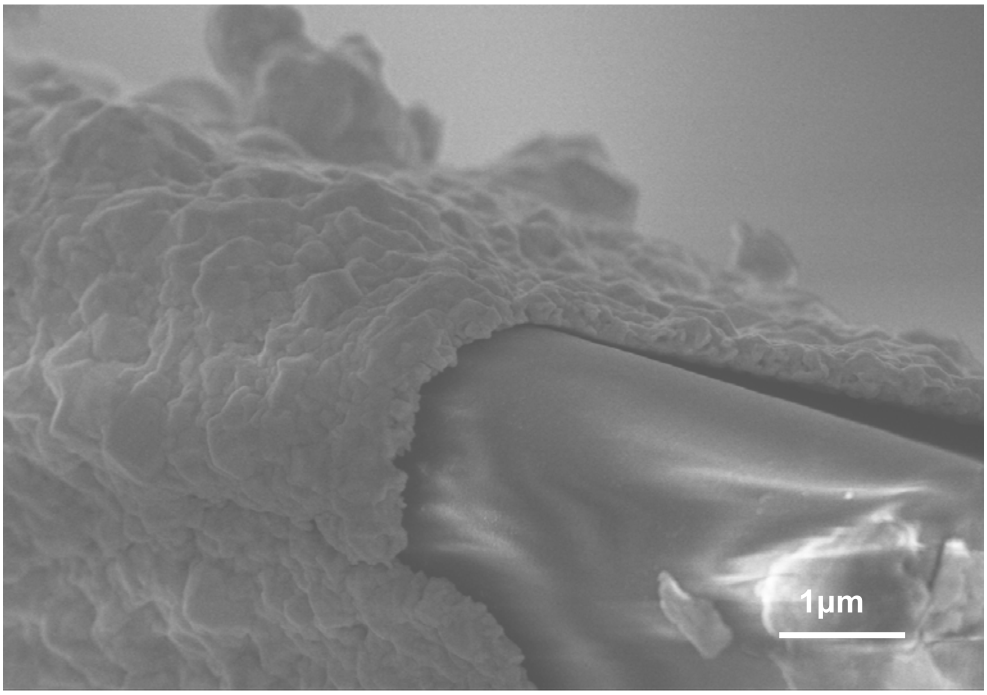

Figure S5. Small interstice between the silver film and the polymer.

Supplementary Figure S6

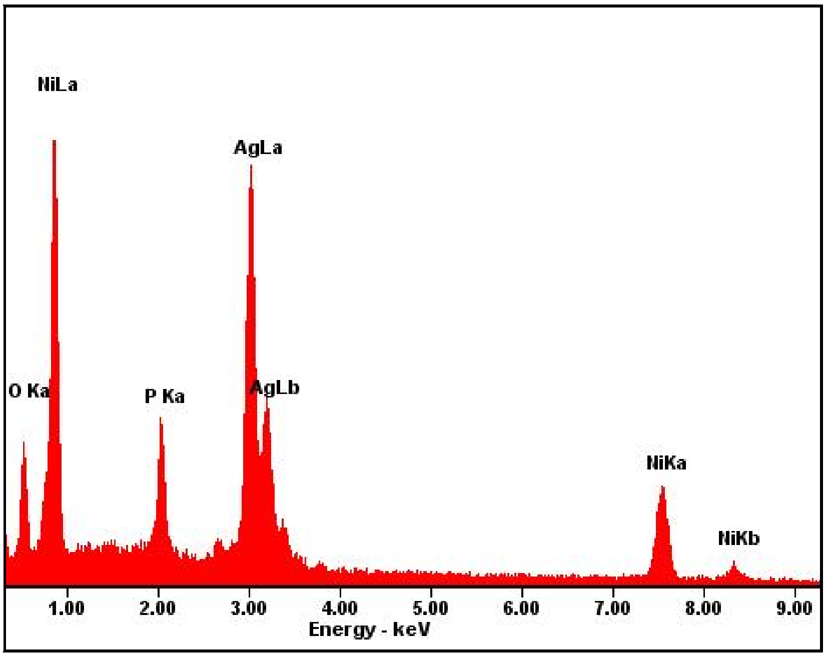

Figure S6. Energy dispersive spectrometer (EDS) analysis of the Ni/Ag foam.

Supplementary Figure S7

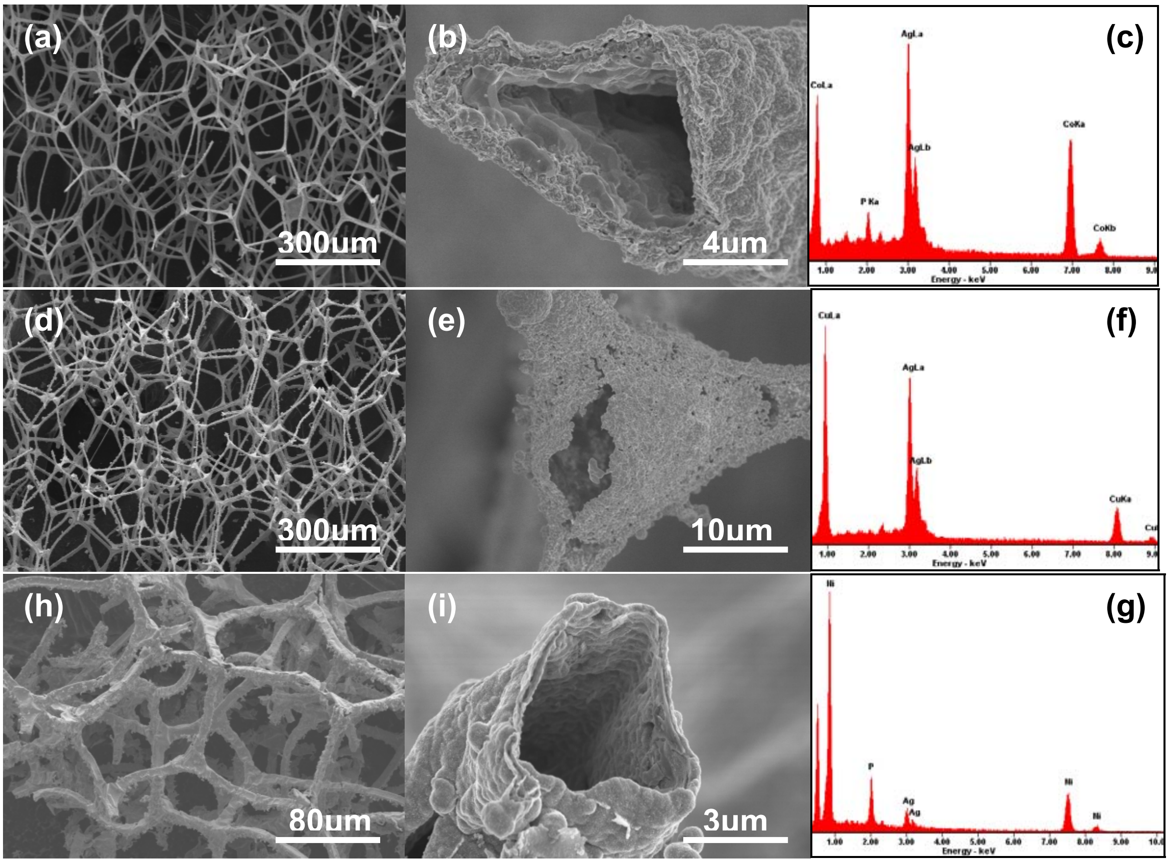

Figure S7. (**a**) (**b**) and (**c**) SEM images and EDS of the Co/Ag foam; (**d**) (**e**) and (**f**) SEM images and EDS of the Cu/Ag foam; (**h**) (**i**) and (**g**) SEM images and EDS of the Ni foam.
